# Quantifying two-dimensional and three-dimensional stereoscopic learning in anatomy using electroencephalography

**DOI:** 10.1038/s41539-019-0050-4

**Published:** 2019-07-17

**Authors:** Sarah J. Anderson, Heather A. Jamniczky, Olave E. Krigolson, Sylvain P. Coderre, Kent G. Hecker

**Affiliations:** 1 Department of Veterinary Clinical Diagnostic Sciences, Faculty of Veterinary Medicine, University of Calgary, Calgary, AB Canada; 2 Department of Community Health Sciences, Cumming School of Medicine, University of Calgary, Calgary, AB Canada; 3 Department of Cell Biology and Anatomy, Cumming School of Medicine, University of Calgary, Calgary, AB Canada; 4 Centre for Biomedical Research, University of Victoria, Victoria, BC Canada; 5 Undergraduate Medical Education Program, Department of Medicine, Cumming School of Medicine, University of Calgary, Calgary, AB Canada

**Keywords:** Perception, Operant learning, Education, Learning and memory

## Abstract

Advances in computer visualization enabling both 2D and 3D representation have generated tools to aid perception of spatial relationships and provide a new forum for instructional design. A key knowledge gap is the lack of understanding of how the brain neurobiologically processes and learns from spatially presented content, and new quantitative variables are required to address this gap. The objective of this study was to apply quantitative neural measures derived from electroencephalography (EEG) to examine stereopsis in anatomy learning by comparing mean amplitude changes in N250 (related to object recognition) and reward positivity (related to responding to feedback) event related to potential components using a reinforcement-based learning paradigm. Health sciences students (*n* = 61) learned to identify and localize neuroanatomical structures using 2D, 3D, or a combination of models while EEG and behavioral (accuracy) data were recorded. Participants learning using 3D models had a greater object recognition (N250 amplitude) compared to those who learned from 2D models. Based on neurological results, interleaved learning incorporating both 2D and 3D models provided an advantage in learning, retention, and transfer activities represented by decreased reward positivity amplitude. Behavioral data did not have the same sensitivity as neural data for distinguishing differences in learning with and without stereopsis in these learning activities. Measuring neural activity reveals new insights in applied settings for educators to consider when incorporating stereoscopic models in the design of learning interventions.

## Introduction

Advances in computer visualization enabling both 2D and 3D anatomical representations have generated tools to aid perception of spatial relationships and provide a new forum for instructional design.^1,2^ To date, studies examining the effectiveness of these educational tools have been comparative, using performance measurements as proxy variables for learning.^3,4^ Furthermore, many of these studies have not been designed to allow for direct comparisons across conditions since implementation of substantially different instructional methods across conditions confounds findings.^5^ In these cases, findings implicating 3D visualizations are invalid without controlling for the influence of instructional methods. While these studies highlight anecdotal enthusiasm for the use of new technology, conclusions typically drawn from such studies imply 3D visualizations are “better” than 2D, or yield no significant differences in learning.^1,6–8^

Beyond anatomy education, 3D visualizations have been investigated in various disciplines particularly in science, technology, mathematics, and engineering (STEM). Much of this work has been conducted by researchers connected with the Spatial Intelligence and Learning Center (https://www.silc.northwestern.edu/) and has explored visuospatial learning from a cognitive perspective. However, a caveat to the abundance of research historically conducted in anatomy and STEM is that technological limitations have necessitated that 3D visualizations be projected on a 2D display. This means that the so-called 3D visualizations achieve understanding of depth through monocular cues.^9^ Monocular cues require only one eye to perceive depth and include cues such as relative size of objects, occlusion, shading, and motion parallax (objects moving at different speeds based on depth).^10^ Now that advances in stereoscopic 3D display technology permit understanding of depth through both monocular and binocular cues, a reexamination of these paradigms is pertinent.^11^ Stereoscopic displays take advantage of humans’ binocular visual system by presenting two images from slightly different views, called “stereo pairs”, to the right and left eyes such that the brain interprets the images as one scene and assigns depth (stereopsis).^9^ Research in other disciplines has revealed that stereoscopic displays are more beneficial when comparing distances, locating or identifying objects, spatially manipulating objects, and navigating.^12^ Finally, a recent study by Wainman et al. in anatomy education compared physical models to match 3D models (projected on a 2D display) to isolate why physical models seem to be superior to computer-based models.^13^ They were able to rule out effects due to learning vs. testing environments as well as the added haptic feedback derived from the ability to touch a physical model as causal factors for superiority. Interestingly, when they covered one eye (thus inducing monocular vision) any advantages of the physical model over the 3D model (projected on the 2D display) were eliminated. Therefore, the presence or absence of stereopsis appears to be a mitigating factor for learner success.^13^

A key knowledge gap in the field of health professional education is the lack of understanding of how the brain neurobiologically processes information from different spatial presentations of content during learning. There is a need for carefully designed studies that isolate stereopsis as the true manipulated variable in the experimental design thus avoiding the methodological inconsistencies identified in prior work.^9,12,14^ By examining the neural mechanisms associated with learning from 2D and stereoscopic 3D content, we may be able to more directly understand differences and implications of stereopsis in learning.

Event related potentials (ERP) measured by electroencephalography (EEG) track learning using reinforcement-based learning paradigms.^15,16^ Two key ERP components of interest are the N250 and reward positivity.

The N250 ERP component is a marker for, and increases in amplitude with acquisition of, visual perceptual expertise.^17–19^ Stated otherwise, greater N250 activity is correlated with heightened visual perception skills. The N250 ERP component produces a negative deflection in the N2 ERP waveform, is thought to be generated in or near the fusiform gyrus, and signal is measured maximally over the posterior area of the scalp.^20–22^ Expertise in facial recognition has historically been one of the most widely studied examples; however, research on discrimination within other specific categories has extended our understanding of processes once thought to be specifically face selective.^17,18,23,24^ A series of experiments by Scott et al. examined the N250 ERP component in a categorization training task using nonface objects including different bird species^17^ and vehicle models.^18^ Expertise in this case is the ability to make use of fine detail information in order to categorize objects at the subordinate level. Scott et al. determined that visual training at the subordinate level (for example, training to identify owl species (snowy owl, burrowing owl, etc.)) rather than the basic level of categorization (for example, to identify a bird as an owl instead another type of bird (owl vs. wading bird)) led to improvements in perceptual expertise for both trained and untrained examples.^17^ They found that the N170 and N250 ERP component mean amplitudes correlated with acquisition of expertise. Specifically, it was hypothesized that the N170 mediates basic-level category or coarse object information (i.e., owl vs. other bird), while the N250 was only sensitive to training at the subordinate level and is modulated by processing of finer detail required for subordinate level discrimination (i.e., snowy owl vs. burrowing owl).^17^ In a follow up study, participants learned to classify cars (sedans, SUVs, and antiques) at the basic or subordinate level.^18^ This work extended observations of the N250, noting increased amplitude is still present 1-week post training in the subordinate level training case and correlates to performance.^18^ Therefore, the N250 ERP component reflects the acquisition of long-term memory for perceptual categorization expertise at the subordinate (more detailed) level.^18^ Increases in the N250, but not N170, as a result of learning in a visual perceptual categorization task focussed on fine detail, have supported the theory that N250 amplitude reflects expertise in subordinate level object recognition.^16^

The reward positivity ERP component is associated with internal evaluation of external feedback during learning.^16,25,26^ The reward positivity ERP component influences the N2 ERP waveform, is sensitive to positive feedback, and represents prediction error, quantifying the difference between actual and expected outcomes when receiving positive feedback.^16,26–28^ In early learning, reward positivity is greatest, pushing the N2 waveform in the positive direction. With increasing learning, the prediction error underlying the reward positivity gets smaller, and the influence of reward positivity on the N2 wave diminishes, making the N2 waveform move in the negative direction. It is believed that the reduction in reward positivity amplitude reflects an underlying learning process. Meaning that the reward positivity measurements will be greatest when active learning is occurring, and will decrease once learning has occurred and the learner is using recall. Our previous work confirmed prior assertions of how these ERP components track with learning, and extended these findings by examining retention and transfer of knowledge 1 week following initial learning.^15^ We demonstrated successful performance and continued maintenance of the diminished reward positivity during retention activities as well as when learners successfully transferred their knowledge to a new context. Moving forward, extending examination of these ERP components using learnable paradigms and real-world contexts will inform neuroscientific understanding as well as educational practices.^15,29^

The purpose of this study was to examine stereopsis in anatomy learning by comparing changes in the amplitude of the N250 and reward positivity ERP components, measured using a reinforcement-based learning paradigm. Based on trial and error with feedback, participants were to learn how to identify and localize neuroanatomical structures while EEG data were recorded. Participants in this study took part in two experimental visits. During the first visit, participants completed the learning task using either 2D, 3D, or a combination of 2D and 3D anatomical models. Approximately 1 month later, all participants completed an identical task that included both 2D and 3D models to assess retention and transfer of knowledge. Importantly, to isolate the role of stereopsis in learning, the only difference in learning activities across experimental groups was whether anatomical models were presented using stereopsis. Interleaved learning has been shown to generate improved scores on final tests of knowledge and is termed the interleaving effect.^30^ This effect is thought to be particularly beneficial when learning to discriminate between concepts that are subtly different since sequential juxtaposition assists learners in distinguishing categories or concepts from one another.^30^ By including a group of participants that learned using both 2D and 3D models rather than just 2D or 3D alone during their initial learning task, we could examine whether interleaving stereopsis generates similar effects in our data as other research on interleaved learning.

The objective of this study is to provide objective evidence comparing the effectiveness of 2D and 3D anatomical models for learning, retention, and transfer, and the effects of interleaved approaches for learning by measuring the N250 and reward positivity ERPs. We predicted that as participants learned to identify and localize anatomical structures, (a) N250 amplitude would increase and remain elevated during retention exercises, thus demonstrating heightened visual perception skills, (b) N250 amplitude would be greater when participants viewed 3D compared to 2D models, given the additional depth cue to facilitate increased object recognition, and N250 amplitude of participants viewing both 2D and 3D models would be similar to that of those viewing 3D models due to exposure to additional depth cues provided through the 3D trials, (c) reward positivity amplitude would decrease as ability to internally evaluate the correctness of responses improved and amplitude would remain diminished with successful retention, (d) learning from 3D models would enable more efficient learning compared to 2D and result in an earlier downward shift of the reward positivity amplitude, and (e) learning from both 2D and 3D models as an interleaved approach will improve long term retention and result in lower reward positivity amplitude during retention tests in this group compared to the 2D and 3D groups.

## Results

### Behavioral analysis

The mental rotations test^31,32^ confirmed that mental rotations abilities did not differ among assigned experimental groups, *F*(2, 59) = 1.18, *p* = 0.32, η^2^ = 0.04. Participants completed identification tests to assess knowledge of cross-sectional brain anatomy before and after each module. There was a significant effect of the timing of these tests on knowledge performance, *F*(2.16, 122.87) = 570.60, *p* < 0.001, η^2^ = 0.91). There were no significant differences among groups in any pre- or post-test scores, *F*(2, 57) = 0.60, *p* = 0.55, η^2^ = 0.02. Module 1 pretest score was low, 2D: 4.72%, 95% CI [1.64, 7.81]; 3D: 1.91%, 95% CI [0, 4.76]; 2D/3D: 4.76%, 95% CI [1.91, 7.62]. Following the module, there was a significant increase in performance on the post-test score (*p* < 0.001), 2D: 80.56%, 95% CI [71.84, 89.27]; 3D: 84.64%, 95% CI [76.57, 92.71]; 2D/3D: 90.24%, 95% CI [82.17, 98.31].

**Fig. 1:**
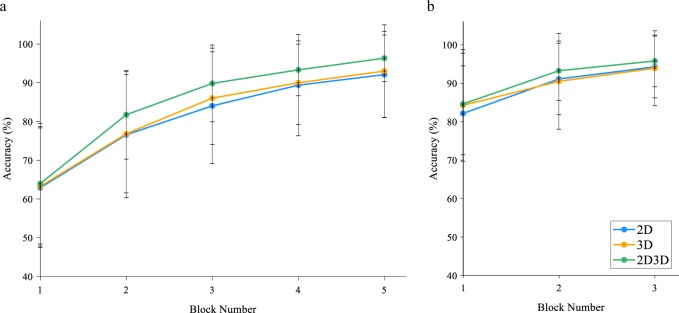
Learning curves showing changes in mean accuracy performance for each block of the modules for all participants. **a** Module 1. **b** Module 2. Error bars indicate ±1 standard deviation

Accuracy performance learning curves were generated by plotting the proportion of correct answers (expressed as a percent) against the block number for each module. The averaged learning curves by condition for all participants are shown in Fig. 1a, and indicated that the proportion of correct responses increased over the five blocks in all conditions. There was a significant effect of block number on neuroanatomical structure identification performance, *F*(3.22, 580.24) = 315.15, *p* < 0.001, η^2^ = 0.64. There were no significant differences among groups, *F*(2, 180) = 3.04, *p* = 0.05, η^2^ = 0.03. Accuracy performance in the first block was as follows: 2D: 62.90%, 95% CI [58.85, 66.94]; 3D: 63.25%, 95% CI [59.41, 67.10]; and 2D/3D: 63.97%, 95% CI [60.12, 67.82]. Mean accuracy improved across each block to the final block where mean accuracy was: 2D: 92.11%, 95% CI [89.50, 94.71]; 3D: 93.02%, 95% CI [90.53, 95.50]; and 2D/3D: 96.35%, 95% CI [93.87, 98.83].

Approximately 30 days following the first module, a knowledge retention test was administered to the participants. There was a significant lapse in knowledge compared to the post-test results of the first module (*p* < 0.001); however, retention performance was still significantly greater than the pretest of the first module (*p* < 0.001). Mean accuracy on the retention test was as follows: 2D: 33.47%, 95% CI [22.28, 44.67]; 3D: 30.30%, 95% CI [19.94, 40.66]; and 2D/3D: 31.13%, 95% CI [20.77, 41.50]. Following the second module, there was a significant increase in performance on the module two post-test score compared to the retention test as well as the post-test of the first module (*p* < 0.05), 2D: 94.03%, 95% CI [88.69, 99.37]; 3D: 92.14%, 95% CI [87.20, 97.09]; and 2D/3D: 97.26%, 95% CI [92.32, 100]. As in the first module, there were no significant differences among groups in the pre- or post-test scores, (*p* > 0.05).

The learning curves generated from the second learning module indicate that the proportion of correct responses increased over the three blocks in all conditions, as shown in Fig. 1b. There was a significant effect of block number on neuroanatomical structure identification performance, *F*(1.63, 284.19) = 113.25, *p* < 0.001, η^2^ = 0.39. There were no significant differences among groups, *F*(2, 174) = 0.86, *p* = 0.42, η^2^ = 0.01. Mean accuracy did not differ (*p* > 0.05) among groups for accuracy performance in the first block of the second module, 2D: 82.13%, 95% CI [78.52, 85.74]; 3D: 84.25%, 95% CI [80.83, 87.67]; and 2D/3D: 84.60%, 95% CI [81.26, 87.94].

However, there was a brief lapse in accuracy performance in the first block of the second module compared to the last block of the first module (*p* < 0.05). Performance in the first block of the second module was similar to module one performance in block 3 in the 2D and 3D groups and block 2 in the 2D/3D group, (*p* > 0.05). Accuracy significantly improved (*p* < 0.05) compared to the first block for the remainder of the second module in all groups.

### EEG data

For each experimental condition, analysis of the N250 and reward positivity were performed independently. A participant’s data were excluded if the electrode site of interest was excessively noisy or >20% of trials were discarded due to artifacts. The number of participants for each analysis was reduced as follows: N250 Module 1 (2D: 15; 3D: 18; 2D/3D: 18), N250 Module 2 (2D: 16; 3D: 20; 2D/3D: 20), reward positivity Module 1 (2D: 18; 3D: 21; 2D/3D: 18), and reward positivity Module 2 (2D: 18; 3D: 20; 2D/3D: 19).

Examination of the ERPs averaged to the onset of presentation of the brain models revealed a bilateral posterior N250 (maximal at channel O1).

**Fig. 2:**
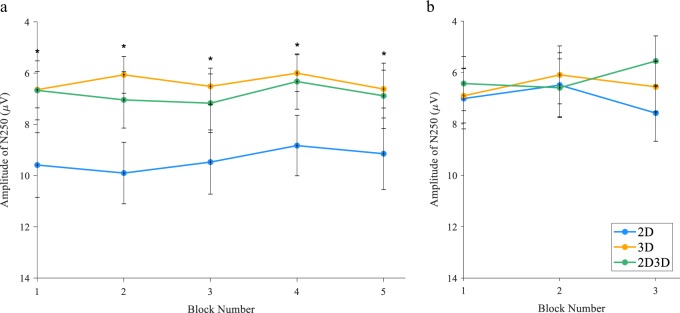
Grand averaged N250 amplitude measured at O1 between 240 and 340 ms. Negative is plotted up. **a** Module 1. **b** Module 2. Asterisk indicates when N250 amplitude in the 3D and 2D/3D groups was significantly more negative (*p* < 0.05, RM_ANOVA, LSD post hoc) compared to the 2D group. Error bars indicate ±95% confidence interval

During the first module (Fig. 2a), there was no effect of block number on N250 amplitude, *F*(3.69, 553.98) = 1.32, *p* = 0.26, η^2^ = 0.01. However, there was a significant difference among groups for N250 amplitude, *F*(2, 150) = 10.96, *p* < 0.001, η^2^ = 0.13. Specifically, the N250 amplitude was significantly more negative (*p* < 0.05) in both the 3D and 2D/3D groups compared to the 2D group in all blocks.

During the second module, all participants completed an identical exercise that employed 2D and 3D models equally over three blocks (Fig. 2b). There was no effect of block number on N250 amplitude, *F*(1.85, 304.50) = 0.95, *p* = 0.38, η^2^ = 0.01, and there was no significant difference among groups, *F*(2, 165) = 0.74, *p* = 0.48, η^2^ = 0.01.

**Fig. 3:**
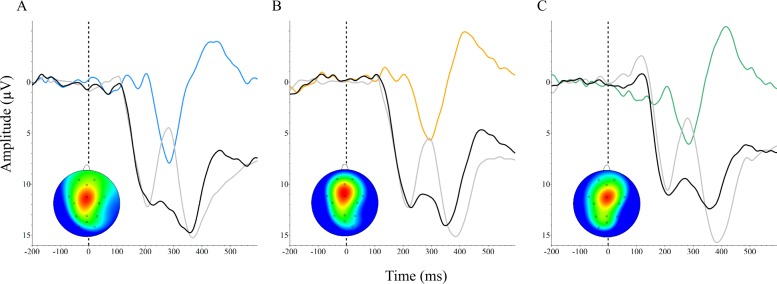
Grand averaged reward positivity ERP waveforms for each group. The ERP waveforms measured at FCz indicate response to correct feedback (black), response to incorrect feedback (light gray), and the difference waveform (colored) and associated scalp distributions calculated by subtracting response to incorrect feedback from correct feedback. Negative is plotted up. **a** 2D. **b** 3D. **c** 2D/3D

Difference wave analysis to confirm presence of the previously reported reward positivity ERP component was performed by comparing neural responses to correct vs. incorrect feedback. Given that participant performance improved in this learning task, difference wave analysis was only possible for the first learning session where there were enough error trials to generate an ERP waveform for response to incorrect feedback. The reward positivity ERP component revealed was consistent with previously reported results and was measured maximally at the FCz channel. The peak latency of the difference wave was 284 ms for 2D, *t*(17) = 5.86, *p* < 0.001, *d* = 1.21; 296 ms for 3D, *t*(20) = 5.18, *p* < 0.001, *d* = 1.06; and 284 ms for the 2D/3D condition, *t*(17) = 6.31, *p* < 0.001, *d* = 0.91 (Fig. 3).

Following confirmation by difference wave analysis, we then extended our analysis across both learning sessions to examine changes in reward positivity amplitudes in response to positive feedback. For this analysis, only trials where participants received correct feedback were considered in each block and a grand average ERP waveform was generated for each block under each condition. Amplitudes of each waveform were compared from 5 ms before to 5 ms after the peak latency.

**Fig. 4:**
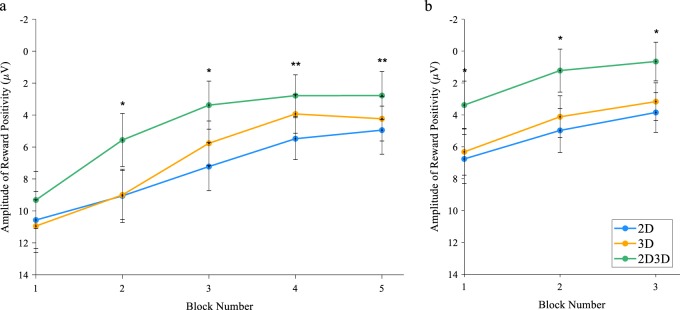
Grand averaged reward positivity ERP amplitude at FCz in response to correct accuracy feedback across all blocks. Negative is plotted up. **a** Module 1. **b** Module 2. Error bars indicate ±95% confidence interval. Asterisk indicates when reward positivity amplitude was significantly more negative in the 2D/3D group than the 2D and 3D groups (*p* < 0.05, RM_ANOVA, LSD post hoc). Double asterisks indicate when reward positivity amplitude was significantly more negative in the 2D/3D group than the 2D group (*p* < 0.05, RM_ANOVA, LSD post hoc)

During the first module, block number had a significant effect on amplitude of response to positive feedback, *F*(3.69, 619.64) = 64.07, *p* < 0.001, η^2^ = 0.28 (Fig. 4a). The amplitude of reward positivity for learners in the 2D group did not differ significantly amongst blocks 1–2 and 4–5 (*p* > 0.05), while blocks 4–5 reward positivity amplitude in the 3D group did not differ significantly amongst blocks (*p* > 0.05), and blocks 3–5 reward positivity amplitude in the 2D/3D group did not differ significantly amongst blocks (*p* > 0.05).

Across all groups reward positivity in response to positive feedback followed a decreasing trajectory, meaning that with learning, participants were less responsive to the feedback. Interestingly, there was a significant difference in reward positivity among groups, *F*(2, 54) = 5.89, *p* = 0.003, η^2^ = 0.07. Reward positivity was similar across all groups in the first block (*p* > 0.05), however reward positivity in the 2D/3D condition was significantly different from both the 2D and 3D conditions in blocks 2 and 3, and from the 2D condition in blocks 4 and 5 (*p* < 0.05).

The amplitude of the reward positivity in the first block of the second module compared to the last block of the first module did not differ in any groups (*p* > 0.05). During the second module (Fig. 4b), block number had a significant effect on amplitude of response to positive feedback, *F*(1.89, 317.97) = 19.81, *p* < 0.001, η^2^ = 0.11. For all conditions, the amplitude of reward positivity was significantly different in the first block compared to the following two blocks (*p* < 0.05).

There was also a significant difference among groups for amplitude of reward positivity in the second module, *F*(2, 168) = 13.40, *p* = < 0.001, η^2^ = 0.14. Specifically, the amplitude of reward positivity was significantly decreased in the 2D/3D groups compared to the other groups in all blocks (*p* < 0.05), and the reward positivity amplitudes did not differ from each other in the 2D and 3D groups (*p* > 0.05).

## Discussion

The research presented here moves beyond assessing enthusiasm and test performance while using 3D visualizations to evaluate the role of stereopsis in anatomy learning by measuring changes in neural activity as a more proximal quantitative dependent variable to compare learning. The objective of this study was to examine stereopsis in anatomy learning by comparing changes in the amplitude of the N250 and reward positivity ERP components measured in a reinforcement-based learning paradigm. We found that participants learning from 3D models had greater object recognition (N250 amplitude) compared to those who learned from 2D models. Second, interleaved learning incorporating both 2D and 3D models provided an advantage in learning, retention and transfer activities represented by decreased reward positivity amplitude.

A key finding of this work is that 2D and 3D neuroanatomical brain models were perceived differently. 3D learning yielded greater object recognition, indicated by the greater N250 amplitude across blocks compared to 2D. This means that stereopsis in the 3D condition may have provided an additional cue that facilitated visual recognition. Despite decreased object recognition when viewing 2D compared to 3D models, there was no significant difference in the efficiency of learning between the 2D and 3D experimental groups from either performance accuracy or reward positivity amplitude perspectives.

Unlike Krigolson et al., we did not observe an increase in N250 amplitude with learning.^16,33^ Since the increases in N250 observed by Krigolson et al. were observed over at least 1000 trials, this may mean that our modules (300 or fewer trials) were too short to observe increases in the N250 amplitude. Despite an observed increase in participants’ anatomical identification skills, the lack of change in N250 amplitude suggests that these participants have not yet developed object recognition skills at the expert level. Experts would be expected to have greater N250 amplitudes compared to these learners, but with greater exposure these learners should demonstrate increases in N250 amplitude. Future work exploring expertise should consider that there may be a threshold below which expertise cannot be distinguished by N250 amplitude and that a minimum number of trials or exposures may be required to observe expertise development.

An alternative explanation for our findings could relate the difference in the N250 amplitude as a result of an increase in perceptual processing demand. For instance, research on the N250 has found increased N250 amplitudes for other-race than own-race faces and found this reflects more effortful processing demands.^34^ With respect to our work, the added element of stereopsis may add processing demand and increase the N250 amplitude as a result. This hypothesis should be explored in future work using analyses associated with working memory while learning.

Despite learner preference for blocked or massed learning designs,^30,35,36^ our work adds quantitative neurological evidence to an increasing body of evidence suggesting that an interleaved learning approach is more beneficial for knowledge retention. Specifically, the combined 2D/3D task conferred a decreased reward positivity amplitude advantage when learners were acquiring skills during the middle of the first module and were assessed for retention in the second module. This finding aligns with prior examples of behavioral-based work showing that interleaved learning improves test scores when distinguishing similarly styled paintings of different artists^37^ and families of birds,^38^ or when learning the appropriate strategies to answer mathematics problems.^39,40^ Interleaved learning may provide advantages over massed or blocked learning since it may draw attention to the differences between categories of models shown thus promoting generalization.^37,41^ The decreased reward positivity amplitude during the middle of the first module and all of the second module may indicate that learners were cross applying information from one dimension to another resulting in a more consolidated memory incorporation. Future research experimental designs incorporating interleaved learning may induce an earlier appearance of changes in the N250 amplitude reflecting changes in visual expertise development.

In this study, we were able to extend examination of reward positivity to a scale more typical of educational studies by using a retention interval duration of ~30 days. Studies of longer retention interval durations have been limited. For example, neuroscientific studies have examined reward positivity on a scale of hours or several days and found maintenance of the reward positivity on recall exercises.^33,42^ Our previous study extended these findings to 7 days and found results similar to Arbel et al.^42^ and Krigolson et al.^33^ but also included intermediate learning exercises that likely strengthened memory recall abilities.^15^

Here, we were able to examine maintenance of reward positivity in the combined 2D/3D group, where learning exercises were the same as the retention exercises. We demonstrated that there was no lapse in reward positivity amplitude at the beginning of the second module compared to the end of the first module despite a lapse in behavioral accuracy performance. Unlike performance accuracy, the reward positivity learning curve did not exhibit a “forgetting curve”.^43^ This means that, despite the lapse in performance accuracy, learners had not lost the ability to internally evaluate their responses and were able to quickly recover performance.

These findings indicate that visual recognition is enhanced when learning using stereoscopic 3D models, which can inform curricular design. Practically, the use of 3D models in teaching should facilitate greater understanding of spatial nuances based on the addition of depth cues. However, exclusive use of 3D models should be avoided. Instead, teaching that includes both 2D and 3D models is likely the most advantageous, as it promotes generalization of knowledge by forcing students to practice moving fluidly between dimensions. To assess the influence of stereopsis in more complex learning environments, carefully controlled experimental designs to understand the interaction amongst variables innate to real educational settings will be required. This research is important since these variables may have an additive effect to learner cognitive load and educators should be conscious of overall cognitive load when designing curricula.^44^

It is expected that since stereopsis adds complexity to a visualization, there may be additional cognitive load experienced by learners while interacting with the models. We also require further research to better understand the critical features of stereoscopic models that learners are using to differentiate structures. In our case, to limit potential prior knowledge of anatomical identification, we chose to teach internal brain anatomy, which necessitated the use of cross-sectional models. While the stereoscopic nature of the 3D models does add depth cues that can be used to differentiate between structures, the area where structures were pinned was planar. This means that some 3D learners could have been focusing on other cues like color or screen location to differentiate structures.

In practice, 3D visualizations allow instructors and learners to interact with models by moving and rotating them to reveal new perspectives of anatomical structures. Since the cognitive processes engaged when viewing stereoscopic 3D virtual environments may more closely approximate real environments,^45^ added animation or rotation may generate advantages when transferring knowledge to real world situations. However, if models are animated or allow for rotation, there is potential that this additional variable may also influence outcomes when exploring stereopsis with regards to cognitive load.^44^ While this work used static models to compare learning with and without stereopsis, we need to better understand how interaction with stereoscopic models may influence cognitive load.

When and how best different types of visualizations should be incorporated into curricula and the frameworks used to present this content should supported by informed pedagogy. This research provides evidence supporting the use of traditional reinforcement learning paradigms to build retainable foundational knowledge in anatomy. Just-in-Time-Teaching (JiTT) modules are opportunities for learners gain foundational knowledge by engaging in learning activities prior to interaction with an instructor.^46^ Incorporating reinforcement-based JiTT activities showing anatomical content from both 2D and 3D perspectives using an interleaved approach would release valuable in-class time for educators to focus on deeper, more complex topics. Since learning in this way entails a high number of retrieval instances over a short time period, consolidation of knowledge into long-term memory could be enhanced. Revisiting a module can further enhance long-term memory retention, as predicted by distributed practice theory.^47^ These benefits are clearly evident here, where an increase in performance on the post-test of the second module compared to the first is observed.

Finally, this work explores differences in learning at a group level despite the fact that we know there are individual differences across learners. This approach means that there is inherent variability within the ERP data across subjects. This variability carries into the grand average waveform thereby requiring greater differences between groups to experimentally detect differences. On one hand, this means that the differences in learning we are observing are the result of significant differences neurological activity, which adds confidence to our findings. However, on the other hand, future work should both increase the number of participants and examine individual learning curves to stratify learners into more homogeneous groups (like low learners and high learners) to determine if this reduces the variability in grand average waveforms.^16^ This may allow us to detect more subtle differences about how stereopsis affects learning across different types of learners.

In this work, we have shown the advantage of using neurological evidence to inform our understanding of the role of stereopsis in learning. Experiments that are carefully designed and make use of neurological variables (like the ones developed here) will be essential to generate new insights about the relationships between visual processing, learning, memory, and cognitive load.

In conclusion, these findings provide new insight for educators to consider when incorporating stereoscopic models in the design of learning interventions. Our results provide quantitative evidence of neurological differences generated while learning from 2D vs. 3D models in anatomy education. Furthermore, our data serve as an exemplar of how the reward positivity ERP component changes with learning, retention, and transfer in an educational setting. Finally, this study demonstrates the feasibility of using a neuroeducational approach to distinguish nuanced differences in learning beyond which behavioral measures alone cannot permit.

## Methods

### Participants

Sixty-one participants (37 females, mean age of all participants = 22.84 years (SD = 5.51) were recruited from health sciences programs at the University of Calgary, Canada. Programs included: Bachelor of Health Sciences, Bachelor of Biomedical Engineering, Bachelor of Biological Sciences, Veterinary Medicine, Medicine, Nursing, and Graduate Sciences Education. Participation was voluntary and informed written consent was obtained in accordance with the Declaration of Helsinki. This study was approved by the Conjoint Health Research Ethics Board at the University of Calgary (Ethics ID: REB14–088).

Participants had minimal neuroanatomical knowledge of cross-sectional brain anatomy, which was confirmed using an identification test prior to the experimental task, thus all recruited participants were included in the study. Participants were randomly sorted into three groups: 2D learning paradigm (19 participants, 12 females), 3D learning paradigm (21 participants, 12 females), and a combined group of 2D and 3D learning (21 participants, 13 females).

Spatial abilities, particularly mental rotations abilities have been shown to be a key predictor of success in learning anatomy.^48–50^ To confirm mental rotations abilities did not differ between groups, as part of the first visit we administered the redrawn 24-item Vandenberg and Kuse Mental Rotations Test,^31,32^ which has been adapted from the Shepard and Metzler Test.^51^ A test question consists of a single Shepard and Metzler-type stimulus presented on the left with four alternative choices provided on the right where two are matches, and a participant must successfully choose which two are matches to get a point. Participants complete 24 questions broken into two sets with 3 minutes to complete each set. The maximum test score possible is 24.^32^

All participants had normal or corrected to normal vision, and no known neurological impairments. Each participant successfully passed a single random-dot stereogram test of stereopsis, which required the participant to detect a Δ1 mm on-screen disparity.

### Experimental design

The experimental design consisted of two experimental visits where participants completed a computer-based learning module meant to teach identification of cross-sectional neuroanatomy. During the visits, participants were seated comfortably in front of a 24′′ 120 Hz LCD monitor with a resolution of 1920 × 1080 pixels, provided a standard USB gamepad to respond, and EEG data were acquired. Participants were administered an identification test prior to and immediately following modules to assess knowledge. The first module was meant to teach participants how to identify neuroanatomical structures in cross section while the second visit was meant to assess retention and transfer of knowledge to models of differing stereopsis. The second experimental visit occurred 29.97 days (SD = 1.80) following the first visit. One participant from the 2D group was unable to attend the second visit; all other participants completed all parts of the experiment.

### Stereoscopic neuroanatomical models

Digital brain models shown as coronal cross sections were created using a cadaveric brain (from the University of Calgary Anatomical Specimens Collection). An example of one of these models is shown in the Supplementary Fig. 1. A sectioned brain was photographed from a 360-degree perspective. Photogrammetry was performed to reconstruct 2D images into 3D mesh and image overlays using Agisoft PhotoScan (Professional Edition, http://www.agisoft.com). The above process was repeated to create nine unique digital models (at increasing frontal cross section depths of the same brain) to enable viewing of all anatomical structures of interest. Next, each 3D mesh was resized and oriented in space to match with the other models and exported using Blender (Version 2.78, www.blender.org). Each of the overlay images of the brain was adjusted to have similar color, brightness, and contrast using Adobe Photoshop CC (Version 2017.0.1). Finally, the meshes, corresponding image overlays, and digital “pins” (to indicate structures of interest) were stored in a database for the module.

To produce stereoscopic disparity, participants were fitted with NVIDIA 3D Vision^®^ 2 goggles that were synchronized to the screen with an infrared tether. For a stereoscopic 3D model, the “right eye” and “left eye” images were generated from the models. These two images were sequentially synchronized with the shuttering of the stereoscopic goggles (at 120 Hz) such that the participant’s right eye always viewed the anatomical model from the “right eye” perspective and the participant’s left eye always viewed the anatomical model from the “left eye” perspective. Like real world binocular vision, participants perceived these models to have stereoscopic depth. However, for a nonstereoscopic 2D model, the monitor remained in stereoscopic mode, and participants viewed two images from the same perspective that were textured onto a plane at monitor-level depth. This enabled all participants to wear the shutter goggles for the duration of the experiment regardless of learning paradigm.

### Reinforcement-based learning module

Questions were presented on the computer monitor during the modules using a customized script created in Presentation^®^ software (Version 18.3, Neurobehavioural Systems, Inc., Berkeley, CA, www.neurobs.com). Modules were designed based on a task adapted from Krigolson et al.^16^ and a detailed description of this task is previously described in Anderson et al.^15,52^ Briefly, participants were trained to identify and localize neuroanatomical structures over a series of trials (questions) where labeled neuroanatomical models were shown and participants were required to answer whether a label for a structure was correct or incorrect. Through training, participants were expected to improve their accuracy in identifying neuroanatomical structures through trial and error, accompanied by positive and negative feedback based on the accuracy of their responses. The following neuroanatomical structures were used: amygdala, caudate nucleus, cingulate gyrus, corpus callosum, hippocampus, hypothalamus, internal capsule, globus pallidus, putamen, and thalamus. For each structure, three incorrect labels were purposely selected to serve as proximal distractors from the other labels such that all ten structure labels were equally used. For each structure the correct label was shown 50% of the time while one of the other three labels was shown the other 50% of the time. A trial consisted of the following components: a fixation cross (400–600 ms); an image of a brain with a pin indicating a structure of interest (1500 ms); a label for the structure was then added (50% chance of being correct) and participants were required to respond indicating whether the label was “correct” or “incorrect” using the gamepad (maximum time allowed 2000 ms); a fixation cross (600–800 ms). Accuracy feedback was then provided to participants in the form of an “✖” for incorrect trials or “✔” for correct trials. Trials lasted ~5 s and were grouped into blocks of 60 trials (~5 min), participants were provided a rest period following each block, and could advance to the next block when ready. Accuracy and response time information were collected for each trial to construct learning curves. On trials where participants were too slow to respond, it was assumed that the participant would have been incorrect and the maximum time allowed for a response (2000 ms) was assumed for analysis purposes.

During each module the anatomical structure shown in each trial was randomized so that each structure was shown in equal distribution across the blocks. This randomization occurs separately for each participant and visit such that no participant sees the same order of questions when they complete the modules, thus avoiding any interference from order effects. The first module consisted of five blocks (300 trials total). During this module participants viewed the anatomical models as 2D or 3D depending on which experimental group they belonged to: 2D, 3D, or combined 2D/3D. The combined group viewed the models randomly as 2D or 3D on a trial-by-trial basis such that each was viewed 50% of the time. During the second visit the module consisted of three blocks (180 trials total) where all participants viewed the models in the combined 2D/3D format. The first learning module was completed over ~30 min, while the second module was ~20 min.

### EEG data acquisition and analysis

We recorded EEG data during the learning modules from 16 electrode locations on the scalp (FP1, FP2, AFz, FC5, FC6, FCz, C3, C4, TP9, P3, Pz, P4, TP10, POz, O1, O2, plus ground, and reference) using an actiCAP Xpress acquisition system arranged in a standard 10–20 layout (Brain Products, GmbH, Munich, Germany) and Brain Vision Recorder Software (Version 1.20, Brain Products, GMbH, Munich, Germany). Electrode impedances were kept below 20 kΩ. EEG data were sampled at a rate of 500 Hz and amplified (V-Amp, Brain Products, GmbH, Munich, Germany: 0–500 Hz bandwidth, 24-bit A/D conversion). A timing correction was applied using a photosensor to realign the stimulus markers and EEG data.

EEG data were processed as in Anderson et al.^15^ using Brain Vision Analyzer 2 software (Version 2.1, Brain Products, GmbH, Munich, Germany). Data were rereferenced offline from a common reference to linked TP9 and TP10 electrodes, and filtered using a phase shift-free Butterworth filter with a 0.1–30 Hz passband and a 60 Hz notch filter. Here we used slightly more stringent parameters for removing artifacts, where a trial was discarded if the voltage on any channel exceeded 10 μV/ms gradient and 100 μV absolute difference criteria.

Epochs for the N250 and reward positivity ERP components were created. Grand average ERP waveforms were generated for each ERP component for each block by averaging EEG epochs across all participants for each experimental condition (2D, 3D, and 2D/3D).

For the N250, the event of interest was the appearance of the brain image. We defined the N250 ERP component as the mean voltage from 240 to 340 ms following presentation of the stimulus at electrode site O1. This latency window was selected based on visual inspection of the grand average waveforms and electrode site was reported for where the N250 amplitude was maximal.^16–19^

For reward positivity, epochs were linked to positive feedback onset and measured maximally at the FCz electrode site (overlying the medial frontal cortex). Since the peak latency of the reward positivity varied across blocks, the latency of the most negative peak between the P200 and P300 waves was identified and the reward positivity was defined as the mean voltage ±5 ms from this peak.

### Statistical analysis

Statistical analysis was performed using SPSS Statistics (Version 24). One-way ANOVA was used to determine if differences in mental rotations abilities based on the assigned experimental groups were present. Paired samples *t*-tests were used to compare the difference between response to positive and negative feedback. Repeated measures analysis of variance (RM-ANOVA) was used to compare behavioral changes (knowledge test performance and block by block accuracy during modules) as well as amplitude changes across the entire experiment, both for within and among experimental conditions for N250 and reward positivity amplitudes. Interaction effects were examined but not reported, as no significant interaction effects were found. Least significant difference (LSD) post hoc analysis was used to identify significant differences. An additional Greenhouse–Geisser correction was applied to adjust the degrees of freedom when Maulchy’s Test of Sphericity indicated that the assumption of sphericity was violated. Effect sizes were determined by calculating Cohen’s *d* (*t*-tests) and partial eta squared (η^2^, RM-ANOVA). An alpha level of 0.05 was assumed for statistical significance in all tests.

## Supplementary information

Supplementary Figure 1

## Data Availability

The data that support the findings of this study are available from the corresponding author upon reasonable request.

## Supplementary information

**Supplementary Information** accompanies the paper on the *npj Science of Learning* website (10.1038/s41539-019-0050-4).

## Code availability

Enquiries about code access can be made to the corresponding author.
