# Supplementary material for: Quantifying two-dimensional and three-dimensional stereoscopic learning in anatomy using electroencephalography
Source: NPJ Sci Learn. 2019 Jul 17;4:10. doi: 10.1038/s41539-019-0050-4 (PMC6637108; doi:10.1038/s41539-019-0050-4)
Supplement: Supplementary file 1 — Supplementary Figure 1 [file 41539_2019_50_MOESM1_ESM.pdf]

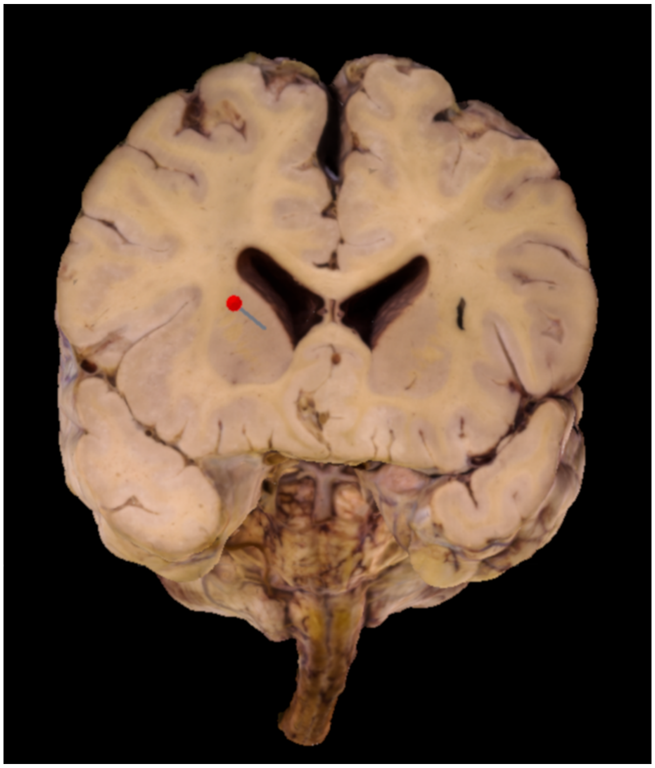

Supplementary Figure 1. Example of a neuroanatomical model used for learning modules. Red pin indicates structure of interest that student learns to identify. Note: models were projected either with or without stereopsis during learning modules.
